# Enhanced perceptual selection of predicted stimulus orientations following statistical learning

**DOI:** 10.1167/jov.23.7.3

**Published:** 2023-07-06

**Authors:** Elizabeth A. Lawler, Michael A. Silver

**Affiliations:** 1 Vision Science Graduate Group and Herbert Wertheim School of Optometry & Vision Science, University of California, Berkeley, Berkeley, CA, USA; 2 Vision Science Graduate Group and Herbert Wertheim School of Optometry & Vision Science, University of California, Berkeley, Berkeley, CA, USA; 3 Helen Wills Neuroscience Institute, University of California, Berkeley, Berkeley, CA, USA

**Keywords:** prediction, expectation, perceptual selection, binocular rivalry, visual statistical learning

## Abstract

Perception is influenced by predictions about the sensory environment. These predictions are informed by past experience and can be shaped by exposure to recurring patterns of sensory stimulation. Predictions can enhance perception of a predicted stimulus, but they can also suppress it by favoring novel and unexpected sensory information that is inconsistent with the predictions. Here we employed statistical learning to assess the effects of exposure to consistent sequences of oriented gratings on subsequent visual perceptual selection, as measured with binocular rivalry. Following statistical learning, the first portion of a learned sequence of stimulus orientations was presented to both eyes, followed by simultaneous presentation of the next grating in the sequence to one eye and an orthogonal unexpected orientation to the other eye. We found that subjects were more likely to perceive the grating that matched the orientation that was consistent with the predictive context. That is, observers were more likely to see what they expected to see, compared to the likelihood of perceiving the unexpected stimulus. Some other studies in the literature have reported the opposite effect of prediction on visual perceptual selection, and we suggest that these inconsistencies may be due to differences across studies in the level of the visual processing hierarchy at which competing perceptual interpretations are resolved.

## Introduction

Creating perceptual interpretations of underdetermined sensory inputs involves inferences, and predictions based on past sensory experience contribute to these perceptual inferences (Chalk et al., 2010; Fischer & Whitney, 2014; Gibson & Radner, 1937; Liberman et al., 2014). Specifically, when sensory information is ambiguous, prior experience can influence which one of multiple possible perceptual interpretations is selected (Brascamp et al., 2007; Chopin & Mamassian, 2012; Denison et al., 2011; Haijiang et al., 2006; Long et al., 1992; Maloney et al., 2005; Pearson & Brascamp, 2008; Sterzer et al., 2008; Wolfe, 1984). Here we investigated the effects of prediction on perception by utilizing statistical learning of sequences of oriented gratings and then testing the influence of the resulting predictions on perceptual selection of visual stimuli in an ambiguous bistable display.

In our study, statistical learning was utilized to create implicit predictions of which grating orientation would appear next in a sequence. Statistical learning is the process of rapidly acquiring representations of regularities in the environment through probabilistic inference (Aslin & Newport, 2012), and it has been demonstrated for patterns of abstract shapes (Fiser & Aslin, 2001, 2002; Turk-Browne et al., 2008), natural images (Brady & Oliva, 2008; Meyer & Olson, 2011), grammatical structure (Saffran, Aslin, & Newport, 1996), and sounds (Gebhart, Newport, & Aslin, 2009; Saffran et al., 1999). Statistical learning does not require allocation of attention to the specific patterns that are being learned, and in fact, individuals are often completely unaware of the patterns that they have learned (Musz, Weber, & Thompson-Schill, 2014; Saffran et al., 1997).

Individuals can unconsciously learn sequential regularities through passive viewing of long streams of stimuli that contain consistent sequences of elements that appear in the same temporal order (Aslin & Newport, 2012; Saffran et al., 1999). This statistical learning can then be assessed using implicit measures (e.g., reduction in response time for expected stimuli) or explicit measures (e.g., asking subjects to judge whether a sequence that had been presented during learning is more familiar than a novel sequence).

In the present study, we measured perceptual selection with binocular rivalry, a bistable perceptual phenomenon that occurs when two incompatible images are presented to the two eyes at overlapping retinal locations. This leads to a perceptual alternation between the competing images, even though the visual stimuli remain constant (Alais & Blake, 2005; Blake & Wilson, 2011). This dissociation between unchanging stimuli and a fluctuating conscious perception provides a powerful paradigm for investigating the influence of contextual factors on visual perceptual selection. Recent work has revealed that diverse factors influence perceptual selection in binocular rivalry, including spatial context (Bressler, Denison, & Silver, 2013), emotional context (Anderson et al., 2011; Sheth & Pham, 2008; Yoon et al., 2009), task relevance (Chopin & Mamassian, 2012), and volitional and attentional state (Dieter & Tadin, 2011; Paffen & Alais, 2011).

Perceptual selection is also influenced by ecological factors and past experiences that may manifest as implicit expectations, or priors, regarding the visual environment. For example, images with natural image statistics dominate over more artificial images (Baker & Graf, 2009), and upright faces tend to dominate over inverted faces (Engel, 1956; Hastorf & Myro, 1959). Moreover, images of floors dominate over images of ceilings (Ozkan & Braunstein, 2009), which may reflect greater sensitivity to ground surfaces over ceiling surfaces (Bian et al., 2005; McCarley & He, 2000) that is based on priors arising from lifelong experience in traversing ground surfaces. The fact that ecological factors influence perception during binocular rivalry indicates that it is a good model for studying how priors contribute to visual perceptual inference. Additionally, binocular rivalry is less susceptible to volitional cognitive control compared to other types of multistable percepts (Liebert & Burk, 1985; Meng & Tong, 2004; Peterson, 1986; Toppino, 2003; Shimono et al., 2011) and is therefore an excellent tool for investigating the effects of predictive context on perceptual selection and early visual processing while mitigating possible effects of response bias.

Influences of priors on perceptual selection in binocular rivalry have been documented for multiple types of stimuli and predictions. For example, in Denison et al. (2011), participants viewed sequences of sinusoidal gratings that generate a percept of apparent rotational motion, thereby creating an expectation that the next grating in the series would continue that direction of rotation. When a grating with the predicted orientation was then presented together with an orthogonal grating in a binocular rivalry display, it was more likely to be initially perceived (Denison et al., 2011). Additionally, statistical learning of arbitrary auditory-visual associations also results in increased likelihood of initial perception of the expected image in binocular rivalry when it immediately follows presentation of its paired sound (Piazza et al., 2018).

In the examples described above, the predicted stimulus is more likely to initially dominate in binocular rivalry—that is, subjects have a tendency to see what they expect to see. However, predictive context generated from learned statistical regularities in sequences of natural images results in the opposite effect on perceptual selection. After statistical learning of arbitrary triplet sequences of images, replaying the first two images of a learned triplet just before presentation of a rivalrous display caused observers to be more likely to perceive the *unexpected* image (i.e., the one that did not match the learned triplet structure) over the expected image (Denison et al., 2016). This suggests that when predictive context is created by more naturalistic stimuli, surprising and novel information may be prioritized for perceptual selection.

One possibility is that the effects of prediction depend on the stage in the visual processing pathways where selection occurs: early visual cortex for simple stimuli such as gratings (Denison et al., 2011; Piazza et al., 2018) and higher-order visual cortex for natural images (Denison et al., 2016). In the present study, we addressed these discrepant results by constraining our predictive context to originate only from visual statistical learning of stimuli of simple features. The study was designed to facilitate comparisons with Denison et al. (2016). Both Denison et al. (2016) and the present study used binocular rivalry to assess the effects of statistical learning of four arbitrary triplet sequences on visual perceptual selection. Denison et al. (2016) employed triplet sequences of natural images for statistical learning, while in the present study, we generated predictions in our participants through statistical learning of triplet sequences of oriented gratings.

In the initial exposure phase, subjects performed a one-back orientation discrimination task on streams of gratings. Unbeknownst to the subjects, these streams contained embedded triplet sequences of specific orientations of gratings that were acquired through statistical learning. After completion of the exposure phase, perceptual selection was assessed by sequentially presenting the first two gratings of each triplet to both eyes to generate an expectation about the next stimulus in the sequence (as was done for triplet sequences of natural images in Denison et al., 2016). This was followed by presentation of a rivalry display in which the predicted grating orientation was presented to one eye and the orthogonal, unexpected grating was presented to the other eye. We found that subjects were initially more likely to perceive the predicted grating in this rivalry display.

## Methods

### Subjects

Data from 76 naive subjects (out of 105 enrolled subjects) were analyzed in this study. Four groups differed in the duration of exposure during statistical learning: 29 subjects in Group 1 (age range 18–27; mean age = 20; 20 female, 9 male), 26 in Group 2 (age range 18–29; mean age = 22; 11 female, 15 male), 27 in Group 3 (age range 18–39; mean age = 22; 19 female, 8 male), and 23 in Group 4 (age range 18–25; mean age = 21; 15 female, 8 male). All subjects provided informed consent, and all experimental protocols were conducted in accordance with the Declaration of Helsinki and approved by the Committee for the Protection of Human Subjects at the University of California, Berkeley.

### Visual stimuli

Stimuli were generated on a Macintosh PowerPC using MATLAB and Psychophysics Toolbox (Brainard, 1997; Pelli, 1997) and displayed on two halves of a gamma-corrected NEC MultiSync FE992 CRT monitor with a refresh rate of 60 Hz at a viewing distance of 100 cm. Subjects viewed all stimuli through a mirror stereoscope with their heads stabilized by a chinrest. Visual stimuli were gratings presented within circular patches 2.5° in diameter that were surrounded by a black annulus with a diameter of 3.6° and a thickness of 0.28°. Binocular presentation of this annulus allowed it to serve as a vergence cue to stabilize eye position and to ensure that rivaling stimuli were presented to corresponding retinal locations in the two eyes. All gratings had a spatial frequency of 2.2 cpd and 10% contrast and were presented on a neutral gray background (luminance = 59 cd/m^2^). During rivalry presentations, one grating was tinted red and the other grating was tinted blue by increasing the intensity in the red and blue channels by 50%.

The set of gratings used for the first and second positions of each triplet contained 10 possible orientations that were grouped into four unique triplet sequences. The first two gratings were drawn from a subset of eight orientations ranging from −75° to +75° in increments of 15°, excluding the vertically oriented grating and ± 45° from vertical. The third grating of each triplet was tilted either 45° to the left or 45° to the right of vertical. The orientations of the gratings in the first and second positions of each triplet were never used for the rivalry stimuli, and the sequences were selected so that there was no perception of apparent rotational motion for the gratings in any of the triplets.

### Eye dominance screening

After enrolling in the study, each observer's eye dominance was measured. On each of 50 trials, subjects viewed a pair of static orthogonal rivalrous gratings with +45° and −45° orientations for 5 s and continuously reported which orientation they perceived using a pair of buttons on a keypad. Eye dominance was defined as the proportion of trials in which the initial percept corresponded to the grating presented to the left or right eye.

### Subject exclusion

Subjects whose initial eye dominance in either eye was greater than 80% during screening were excluded and did not participate further in the study. This exclusion criterion was needed because a large bias in favor of either the left or right eye during binocular rivalry would limit our ability to assess the effects of prediction on perceptual selection. Eye dominance was also measured throughout the rivalry test (see below) by analyzing initial rivalry responses, and subjects with > 80% eye dominance during the rivalry test were also excluded from all analyses. Subjects who responded > 80% in favor of one color (red or blue tint) over the other during the rivalry test were also excluded. Subjects with less than 50% correct trials on the exposure phase were excluded. Finally, subjects were excluded for using incorrect response keys during the rivalry test, as assessed with catch trials in which the same orientation and tint were presented to both eyes (subjects who performed lower than 60% on these catch trials were excluded).

For Group 1, 10 subjects out of 29 (38%) were excluded: 1 subject for poor performance in the exposure phase, 5 subjects for eye dominance, 1 for poor performance on catch trials, and 4 for eye dominance and poor performance on catch trials. For Group 2, 6 subjects out of 26 (23%) were excluded: 3 for eye dominance, 1 for poor performance on catch trials, and 2 for color bias during the rivalry test. For Group 3, 7 out of 27 (26%) subjects were excluded: 5 for eye dominance and 2 for color bias in the rivalry test. For Group 4, 3 subjects out of 24 (12.5%) were excluded for eye dominance.

### Exposure phase

Each of four experimental groups was exposed to the triplet structures for variable durations: Group 1: two exposure blocks/40 presentations of each triplet (approximately 10 minutes of exposure); Group 2: three exposure blocks/60 presentations of each triplet (approximately 15 minutes); Group 3: four exposure blocks/80 presentations of each triplet (approximately 20 minutes); and Group 4: five blocks/100 presentations of each triplet (approximately 25 minutes).

**Figure 1.**
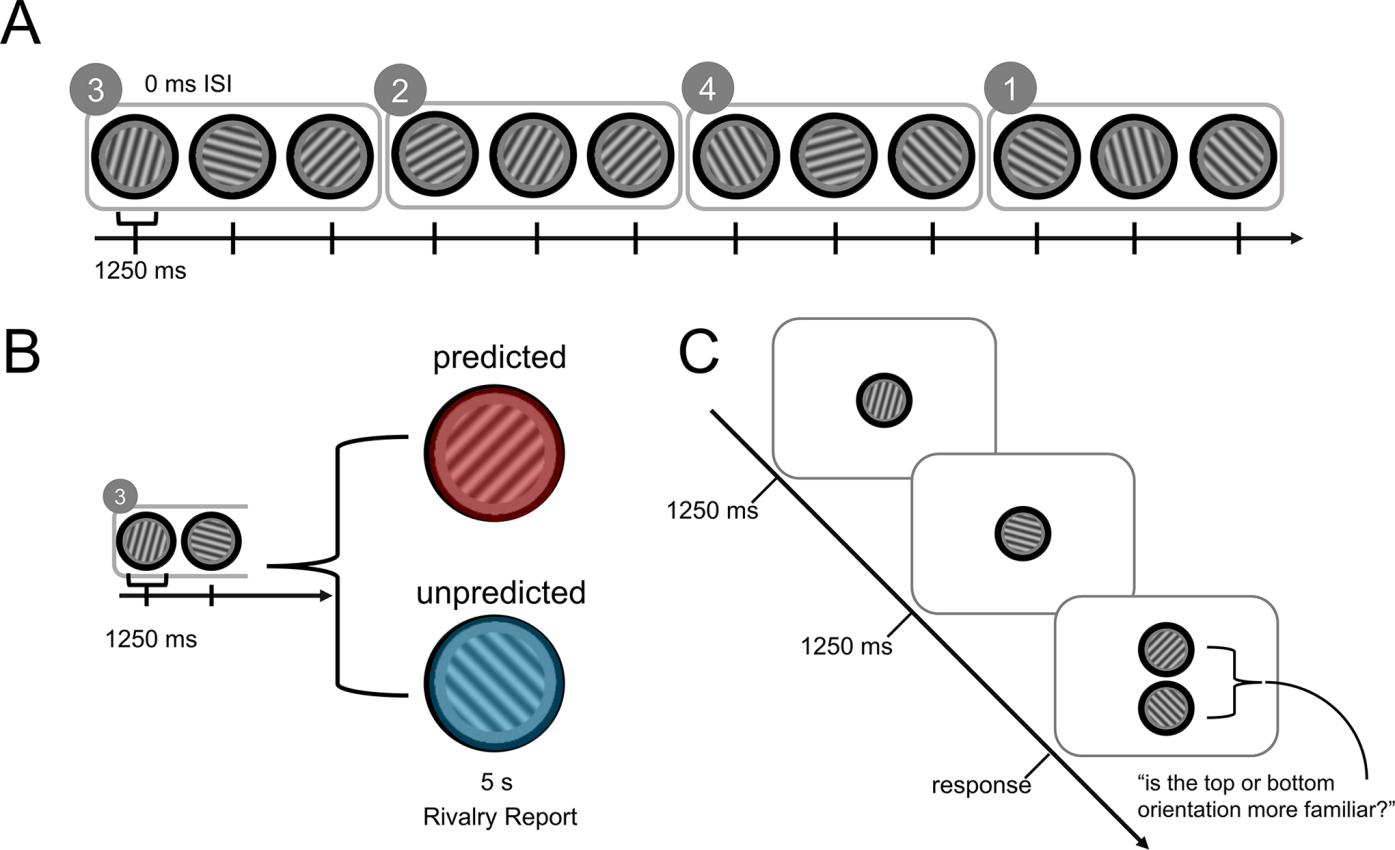
Behavioral tasks. (A) Exposure phase: Subjects viewed a grating stream composed of 10 unique orientations grouped into four triplet sequences (triplet numbers shown in circles) that were presented in pseudorandom order. Each triplet sequence ended in either a left (−45°) or a right (45°) tilt. (B) Binocular rivalry test: Each rivalry display of dichoptic orthogonal gratings was preceded by the first two gratings from one of the triplets, causing one of the rivalrous orientations to be predicted following the sequence context, with the other orientation that did not follow the sequence being unpredicted. The circled number corresponds to the triplet shown in panel A. (C) Familiarity test: On each trial, the first two gratings in one of the triplets from the exposure phase were presented, followed by a pair of gratings. Subjects then were asked to report which of two orientations completed the triplet seen during the exposure phase (2-alternative forced choice). One of the gratings was consistent with the learned triplet, while the other grating orientation was orthogonal to the learned orientation.

A stream of grayscale gratings was presented identically to the two eyes through a mirror stereoscope. Within this stream, all gratings were part of triplet sequences embedded in the stream (Figure 1A). Each grating was presented for 1,250 ms with a 0-ms interstimulus interval. The presentation of the triplets within the stream was pseudorandomized, with the constraint that no triplet could be presented twice in a row within the stream. This constraint reduced the likelihood that subjects would discover the triplet structure within the grating stream during the exposure phase. There were no explicit cues to indicate to the participants that the stream was composed of triplets. This use of arbitrary triplet sequences of oriented gratings established predictive priors while avoiding any previous associations subjects may have had about relationships among the stimuli.

Subjects performed a one-back task in which they compared the orientation of the current grating to that of the previous grating. They were instructed to press one of two keys based on whether the orientation of the current grating was closer to or farther from vertical, compared to the previous grating.

### Rivalry test

Following the exposure phase, each participant completed a rivalry test to assess the effects of statistical learning of the sequences of grating orientations on subsequent visual perceptual selection. On each trial of the rivalry test, the first two gratings from one of the four triplets were first presented identically to both eyes, with the same timing as in the exposure phase (1,250 ms stimulus duration) (Figure 1B). This was immediately followed by a 5-s rivalry display in which the grating presented to one eye was the third grating in the triplet (and therefore predicted by the first two gratings), and the orientation of the grating presented to the other eye was orthogonal and therefore inconsistent with the preceding predictive context.

On all trials, the rivalrous stimuli were grating pairs with 45° and −45° orientations, tinted red and blue (one color in each eye). Subjects reported their percept based on color, thereby making stimulus orientation irrelevant to explicit response selection during the rivalry test. Color tinting also enhanced unitary perception of the rivalry displays. Subjects were instructed to hold down a key for as long as the corresponding percept persisted and to not press any key for periods of ambiguous perception (the absence of unitary perception of either red or blue). The 5-s rivalry duration was usually sufficiently long to generate one unitary rivalry percept followed by at least one perceptual switch to the other orientation, thereby enabling measurement of the duration of initial response on the majority of trials. Subjects completed two runs of the rivalry task. Each run consisted of 96 trials, presented in blocks of 32 trials. Tint colors, left and right eye presentations, and orientations for the gratings in the rivalry display were all counterbalanced. We measured the initial percept (predicted vs. unpredicted grating orientation) as well as the latency and duration of these initial rivalry responses for both predicted and unpredicted percepts.

### Familiarity test

Following the rivalry test, subjects were informed that each grating that was presented during the exposure phase had been part of a sequence of three gratings. Subjects were asked if they had noticed any kind of pattern during the exposure phase, and if they had, they were then asked to describe the pattern they had seen. Next, subjects performed a 2-alternative forced choice familiarity test to measure statistical learning that had taken place during the exposure phase.

Stimuli in the familiarity test were sequences of gratings that were identical to the triplets used in the exposure phase, except for the addition of a second grating that was presented adjacent to the third grating of the learned triplet and had an orthogonal orientation (Figure 1C). Subjects reported which of the two gratings completed the triplet sequence that had been presented during the exposure phase. The timing of each triplet presentation was the same as in the exposure phase, except that the two final gratings remained on the screen until the subject made a response. Each subject completed 32 trials of the familiarity test.

## Results

### Statistical learning of sequences of orientations

**Figure 2.**
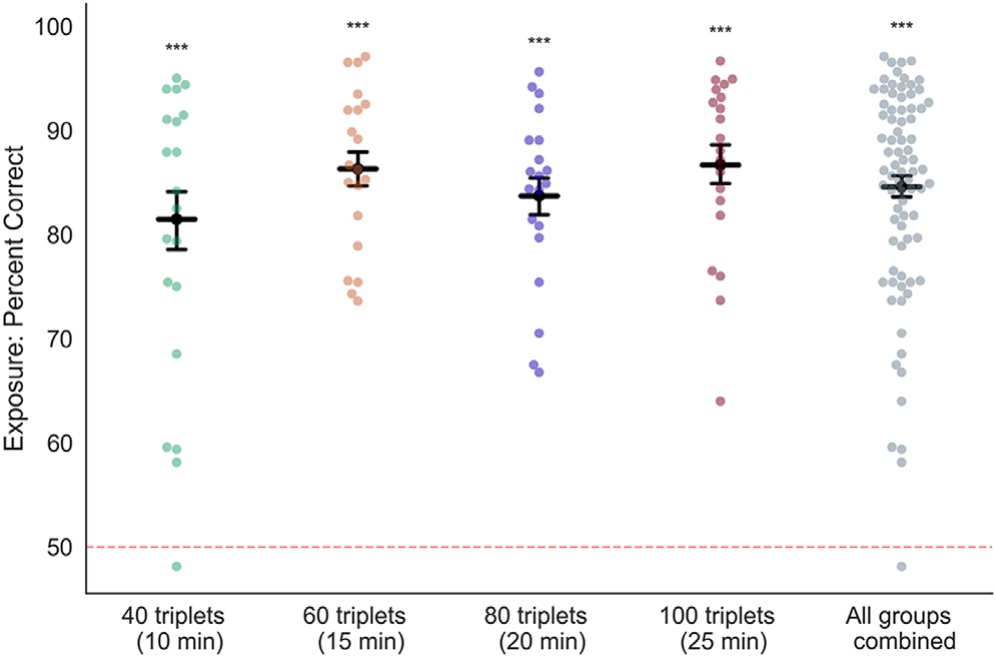
One-back performance during the exposure phase was significantly above chance. Subjects performed well above chance (50%) on the exposure phase (Figure 1A). Dark bars are group averages, and points are individual participant data. Error bars are *SEM* across subjects; *n* = 20 subjects in each condition, *n* = 80 for all conditions combined. ***p < 0.0005. Subjects with performance below 50% correct trials were excluded from further participation.

In the exposure phase, statistical learning of triplets of oriented gratings occurred while each participant performed a one-back orientation task (Figure 1A). For each stimulus in a continuous stream of gratings, subjects reported which of two orientations (current vs. immediately previous grating) was closer to vertical. The length of exposure varied across each experimental group: Group 1 subjects were exposed to a total of 40 repeats of each of four triplets; Group 2, 60 repeats; Group 3, 80 repeats; and Group 4, 100 repeats. Overall accuracy during the exposure phase was 84% (*SD* = 10%), a value that was well above chance performance (50%) but not at ceiling, indicating that the task required subjects to be engaged and attentive (Figure 2). Accuracy was similar for all groups (mean percent correct across subjects, Group 1: 81% [*SD* = 12%]; Group 2: 85% [*SD* = 8%]; Group 3: 83% [*SD* = 7%]; and Group 4: 85% [*SD* = 7%]).

### Binocular rivalry test

After completion of the exposure phase, subjects performed a binocular rivalry test to assess the influence of triplet sequence learning on perceptual selection. Each trial began with sequential presentation of the first two gratings from one of the four triplets from the exposure phase. This was immediately followed by presentation of a pair of rivalrous gratings of orthogonal orientations, one of which always matched the third grating in the triplet (the predicted orientation) (Figure 1B). Subjects reported which of the two orientations they perceived. Based on previous studies (Denison et al., 2011; Denison et al., 2016; Piazza et al., 2018), we expected that the effects of prediction would be largest at the beginning of each rivalry presentation, when predictive context is strongest. We therefore analyzed the initial response following the presentation of the rivalrous pair.

**Figure 3.**
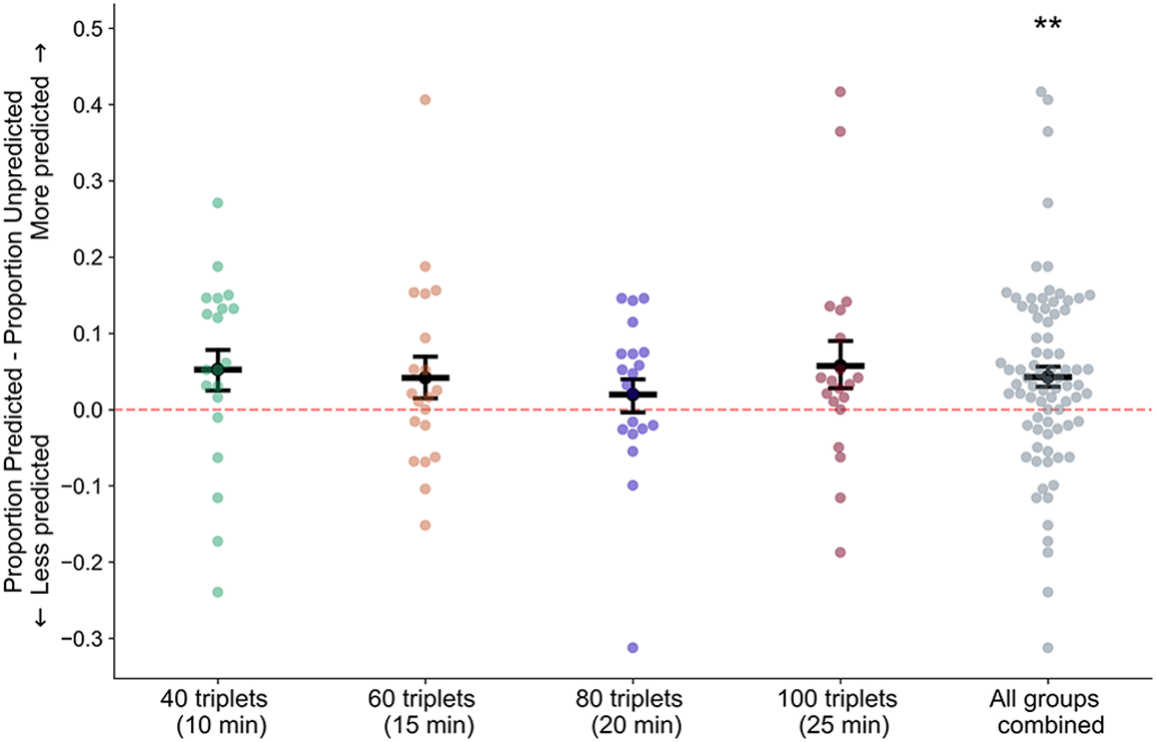
Differences between proportion of trials in which the predicted orientation was initially perceived and the proportion of trials in which the unpredicted orientation was initially perceived. Dark bars represent mean differences, and points represent data from individual participants. Error bars are *SEM* across subjects; *n* = 20 subjects in each condition, *n* = 80 for all conditions combined. **p = 0.001.

In order to investigate the effects of prediction on initial perceptual selection, we first tested for an overall effect of prediction, comparing the proportion of trials in which the predicted grating was initially perceived during the rivalry test to chance (0.5) across all subjects in all groups. We found that across all subjects, initial perception during rivalry was greater for the predicted orientation than for the unpredicted orientation (mean = 0.52, *SEM* = 0.0071, *t*(79) = 3.28, *p* = 0.002, Cohen's *d* = 0.69). Figure 3 shows the difference between the proportion of trials in which the predicted versus unpredicted orientation was initially perceived.

The magnitude of this effect (the predicted orientation was initially perceived on 52% of trials) is very similar to other studies of the effects of prediction on perceptual selection in binocular rivalry. The unpredicted image was selected on 52.6% of binocular rivalry trials following statistical learning of sequences of natural images (Denison et al., 2016), and the predicted stimulus was selected on 52.5% of binocular rivalry trials following statistical learning of pairs of auditory and visual stimuli (Piazza et al., 2018). Natural scenes containing embedded incongruous objects (which presumably violated subjects’ priors about the content of natural scenes) were selected 53% of the time compared to natural scenes that were congruous (Mudrik, Deouell, and Lamy 2011).

Next, we investigated if the length of exposure influenced the effects of prediction on perceptual selection and whether these effects of prediction were maintained throughout the rivalry test. We previously found that the predictive effects of statistical learning on perceptual selection in binocular rivalry can be fleeting, occurring in the first half of the rivalry test trials but dissipating by the second half (Denison et al., 2016). We conducted a two-way mixed-model analysis of variance (ANOVA) with two factors: four levels of group (four experimental groups with different durations of exposure) and two levels of run (each run consisting of the first half and second half of the rivalry test respectively). Effect sizes were quantified with partial eta-squared values. We examined the influence of these two factors on the likelihood of an observer perceiving the expected orientation. We found no significant effects of either group (*F*(3, 76) = 0.46, *p* = 0.71, η^2^_p_ = 0.019) or run (*F*(1, 79) = 0.24, *p* = 0.63, η^2^_p_ = 0.033) and no significant interaction between group and run (*F*(3, 76) = 1.30, *p* = 0.28, η^2^_p_ = 0.051).

We also assessed the effects of prediction on the mean latency and duration of the initial response. The duration of each trial was 5 s, allowing measurement of a complete first response on the majority of trials: 71% of trials had an initial response that terminated before the end of the trial. Unlike the analysis of the identity of the initial response (expected vs. unexpected orientation) presented above, initial response latencies and durations can be analyzed separately for expected versus unexpected orientation responses.

For initial latency and duration, we investigated the effects of prediction on initial perceptual selection with a three-way ANOVA that had four levels of group (four experimental groups with different durations of exposure), two levels of run (each run consisting of the first half and second half of the rivalry test, respectively), and two levels of prediction (reported grating was either expected or unexpected).

**Figure 4.**
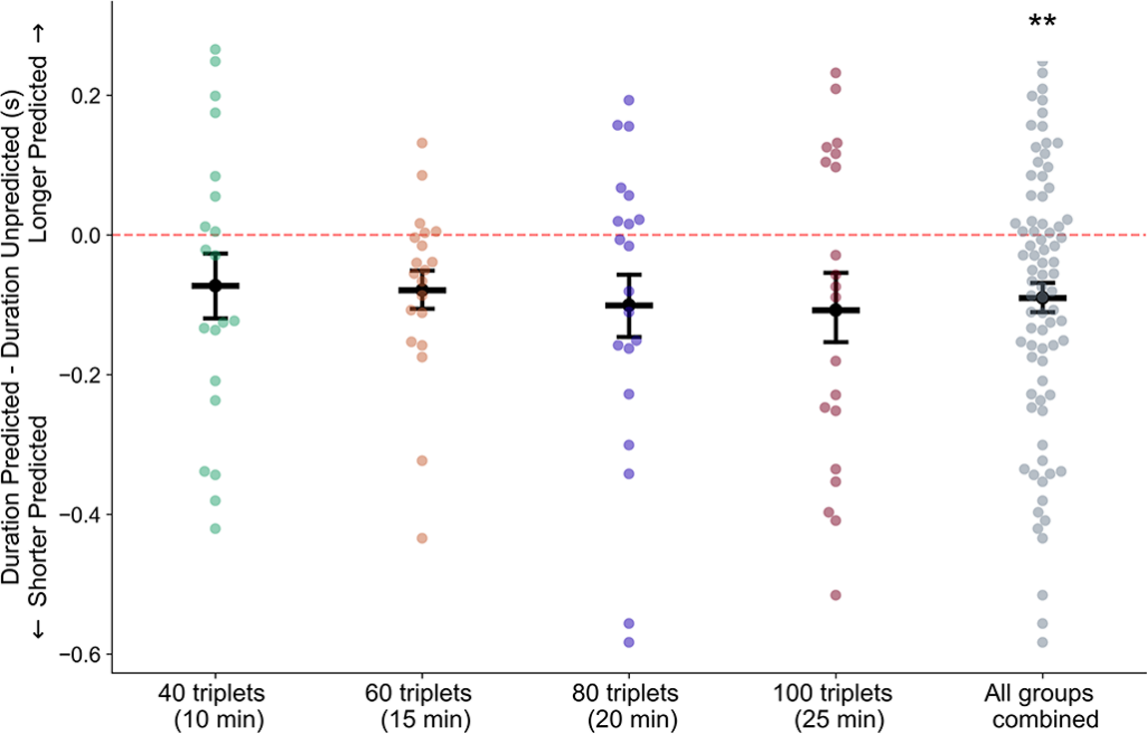
Mean durations of initial responses were shorter for trials in which the predicted grating orientation was perceived. Dark lines represent mean differences in duration between initial percepts of predicted versus unpredicted gratings, and points represent data from individual participants. Error bars are *SEM* across subjects; *n* = 20 subjects in each condition, *n* = 80 for all conditions combined. **p = 0.007.

We found a significant effect of prediction on the duration of the initial response (Figure 4; main effect of prediction, *F*(1, 79) = 19.25, *p* < 0.05, η^2^_p_ = 0.22), such that the initial duration of the predicted grating (mean = 2,390 ms, *SEM* = 49 ms) was significantly shorter than the initial duration of the unpredicted grating (mean = 2,486 ms, *SEM* = 49 ms) (Cohen's *d* = 0.16). The effects of run and group were not significant (run: *F*(1, 79) = 0.66, *p* = 0.42, η^2^_p_ = 0.006; group: *F*(3, 76) = 1.81, *p =* 0.15, η^2^_p_ = 0.075). Additionally, there were no significant interactions between any of the factors (interaction between prediction and run: *F*(1, 76) = 0.37, *p* = 0.54, η^2^_p_ = 0.009; prediction and group: *F*(3, 76) = 1.84, *p* = 0.91, η^2^_p_ = 0.005; group and run: *F*(3, 76) = 1.31, *p* = 0.28, η^2^_p_ = 0.045; prediction/group/run: *F*(3, 76) = 1.58, *p* = 0.2, η^2^_p_ = 0.068). The relatively short rivalry stimulus presentation duration that we employed did not allow for analysis of responses that occurred after termination of the initial percept.

**Figure 5.**
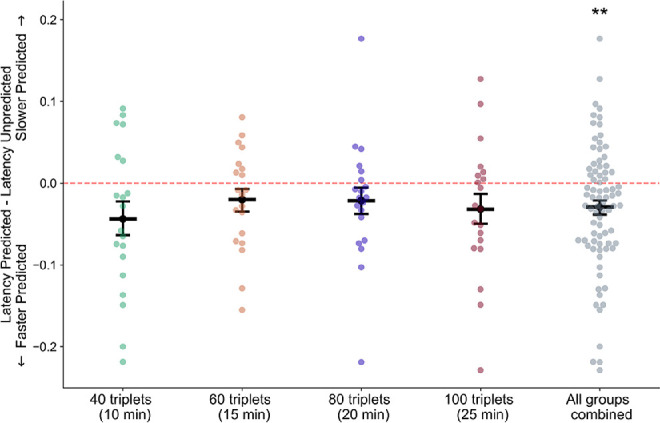
Mean latencies of initial responses were shorter for trials in which the predicted grating orientation was perceived. Dark bars represent mean differences in latency between initial percepts of predicted versus unpredicted gratings, and points represent data from individual participants. Error bars are *SEM* across subjects; *n* = 20 subjects in each condition, *n* = 80 for all conditions combined. **p = 0.002.

We additionally found a significant effect of prediction on the latency of the initial response (Figure 5; main effect of prediction *F*(1, 79) = 9.90, *p* = 0.002, η^2^_p_ = 0.096), such that the initial latency of the predicted grating (mean = 996 ms, *SEM =* 21 ms) was significantly shorter than the initial latency of the unpredicted grating (mean = 1,026 ms, *SEM* = 22 ms) (Cohen's *d* = 0.11). There was also a main effect of run (*F*(1, 79) = 4.38, *p* = 0.04, η^2^_p_ = 0.083), such that the rivalry responses in the first run of trials had a longer latency (mean = 1,028 ms, *SEM* = 21 ms) than those in the second run of trials (mean = 994 ms, *SEM* = 22 ms). The effect of group was not significant (*F*(3, 76) = 0.039, *p =* 1.0, η^2^_p_ = 0.002). There were no significant interactions between any of the factors (interaction between prediction and run: *F*(1, 79) = 1.75, *p* = 0.19, η^2^_p_ = 0.021; prediction and group: *F*(3, 76) = 0.35, *p* = 0.79, η^2^_p_ = 0.003; group and run: *F*(3, 76) = 0.85, *p* = 0.47, η^2^_p_ = 0.031; prediction/group/run: *F*(3, 76) = 0.848, *p* = 0.2, η^2^_p_ = 0.035). In conclusion, the predicted orientation was more likely to be initially selected, and both the mean latency and mean duration of these initial responses were shorter for the predicted orientation.

### Familiarity test

In addition to determining the effects of prediction on perceptual selection in binocular rivalry, we assessed statistical learning for each participant with a familiarity test and a verbal recall interview. In the familiarity test (Figure 1C), subjects were presented with the first two gratings from one of the triplets from the exposure phase, followed by simultaneous presentation of a pair of gratings. One of these gratings in this pair had an orientation corresponding to the third element of the learned triplet, and the other had the orthogonal orientation. Subjects were asked to report which of the two orientations completed the previously seen triplet. Following the task, subjects were interviewed about their ability to explicitly recall any triplets or patterns from the exposure phase.

**Figure 6.**
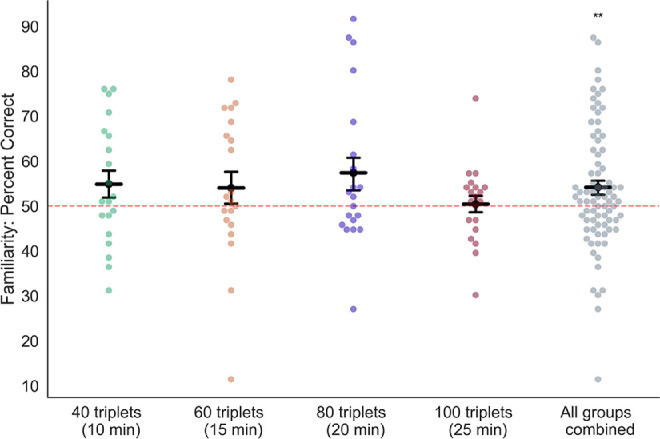
Performance on the familiarity test was significantly above chance. Subjects performed above chance (50%, marked by the dashed line) on the familiarity test (Figure 1C). Dark bars are group averages, and points are data from individual participants. Error bars are *SEM* across subjects; *n* = 20 subjects in each condition, *n* = 80 for all conditions combined. **p < 0.005.

Performance on the familiarity test was significantly above chance (one-sample *t*-test: *t*(78) = 3.35, *p* < 0.005, Cohen's *d* = 0.30) and did not differ across the four exposure duration groups (one-way ANOVA, *F*(3, 76) = 0.81, *p* = 0.49, η^2^_p_ = 0.031), indicating that overall, subjects demonstrated learning of the triplet sequences (Figure 6). However, the relatively poor performance on this task (mean percent correct = 54.2, *SEM* = 1.59) indicates that the amount of explicit learning of triplet sequences during the exposure phase was quite limited.

**Figure 7.**
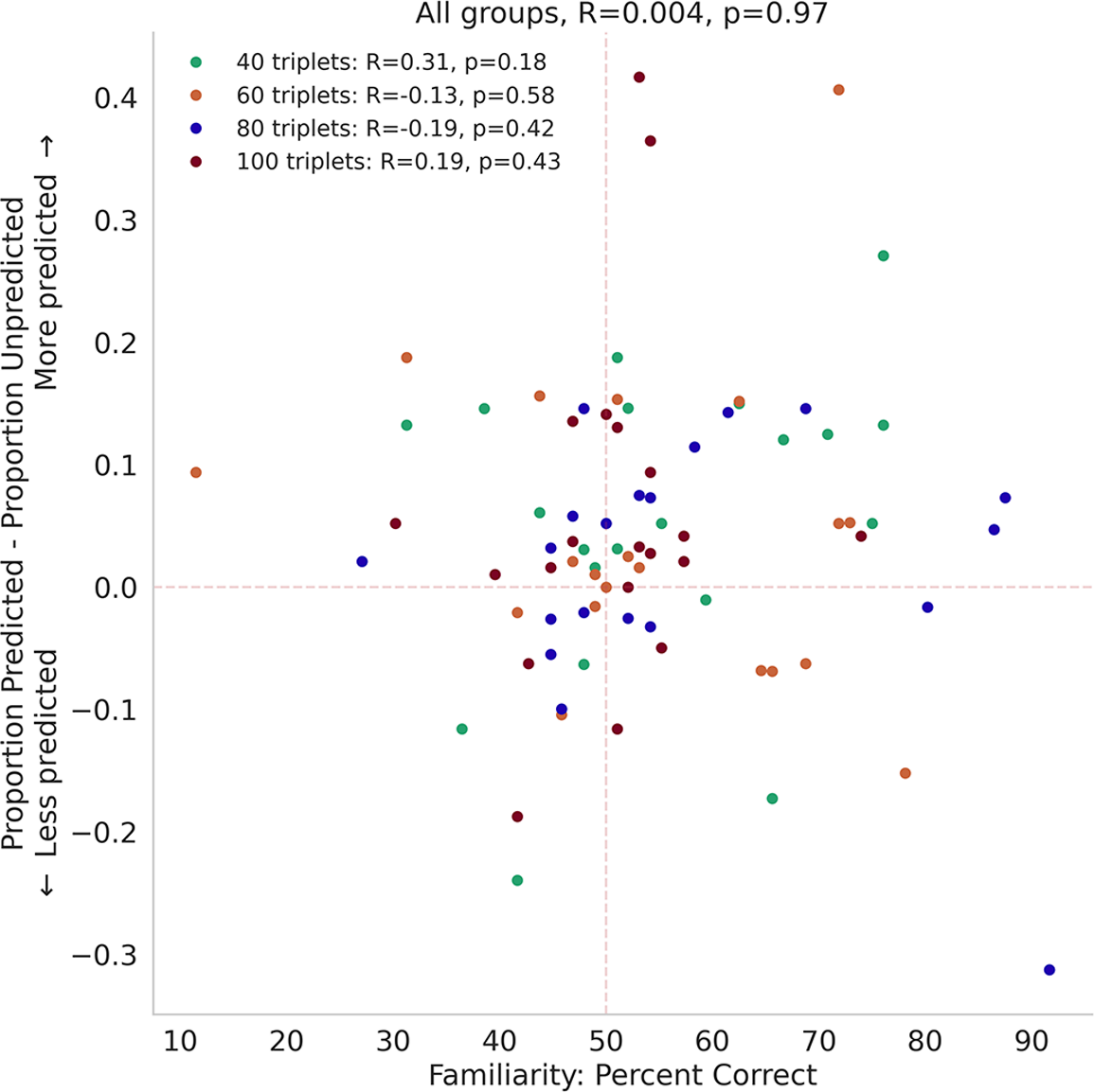
No significant correlation between effects of statistical learning on initial percepts in the rivalry test and on performance on the familiarity test. Subjects’ tendencies to initially perceive the predicted stimulus did not significantly correlate with their performance on the familiarity test, *r*(78) = 0.0040, *p* = 0.97.

Even though there were significant effects of statistical learning on perceptual selection in binocular rivalry and on performance on the familiarity test, the magnitudes of these two effects were not significantly correlated across participants (Figure 7). Additionally, when asked directly if they were aware of any patterns in the exposure phase, only 17 subjects (22.5%) (4 [20%] from Group 1, 3 [15%] from Group 2, 5 [25%] from Group 3, and 5 [25%] from Group 4) verbally reported recognizing any kind of sequential patterns. Of these individuals, four (one from Group 1, two from Group 2, and one from Group 3) were only able to describe sequential patterns of button-press responses from the exposure phase, with no recall of the sequences of the orientations that they saw. Moreover, only the individual in Group 3 identified a correct sequence of button presses, and the rest of the recollections were incorrect. Of the subjects who reported being aware of a pattern, only one subject in Group 1 and two subjects in Group 4 were able to accurately describe one full triplet of orientations. Eight subjects were able to report two of the three orientations in a single triplet (one from Group 1, one from Group 2, four from Group 3, and two from Group 4). Overall, subjects had minimal explicit recall of the sequences of stimuli that were presented to them during the study.

## Discussion

### Effects of predictive context on perceptual selection of oriented gratings

We found enhanced perceptual selection for stimuli that were congruent with a predictable sequential structure that had been acquired by subjects through statistical learning. Specifically, following exposure to arbitrary but consistent sequences of three oriented gratings, when subjects subsequently viewed rivalrous pairs of orthogonal gratings, they were more likely to perceive the orientation that was predicted from a learned sequence compared to an unexpected orthogonal orientation. We found no significant effect of the length of exposure (the duration of time spent learning sequences) on subsequent perceptual selection. This suggests that statistical learning was established at the shortest exposure duration of 10 min and did not substantially increase for longer exposure durations.

### Enhancement of expected versus unexpected stimuli

Our current findings are consistent with studies that have shown perceptual enhancement of expected stimuli in ambiguous displays. Visual imagery (Pearson et al., 2008), learned cue associations (Haijiang et al., 2006; Sterzer et al., 2008), predictable sequences of dot motion (Maloney et al., 2005), action–effect associations (Dogge et al., 2018), task-relevant stimuli (Gayet et al., 2015), cross-modal auditory/visual statistical learning (Piazza et al., 2018), and predictive rotational motion (Attarha & Moore, 2015; Denison et al., 2011) have all been shown to bias perception of ambiguous displays in a direction that is consistent with expectations derived from the accompanying context. These findings support the view that recent visual history can be used to generate predictions that combine with incoming sensory information to increase the likelihood of perception matching the predictions (Bressler et al., 2013; Denison et al., 2011; Kersten et al., 2004).

However, a previous study (Denison et al., 2016) showed the opposite effect: expectations generated through statistical learning of natural images resulted in perceptual enhancement of the unexpected stimulus, relative to the expected stimulus, during binocular rivalry. Both lower-order (Alink et al., 2010; Kok et al., 2012; Richter et al., 2018) and higher-order (Egner et al., 2010; Meyer & Olson, 2011; Richter et al., 2018) visual cortical areas exhibit smaller responses to expected stimuli compared to unexpected stimuli (reviewed in de Lange et al., 2018). Within a predictive coding framework, these expectation suppression/surprise enhancement effects are thought to reflect prediction error, or the degree of mismatch between the prediction and the sensory information. This framework could account for the findings of Denison et al. (2016), but it does not account for our current findings, in which statistical learning of sequences of grating orientations resulted in the expected stimulus dominating perception of a bistable display.

Other than the nature of the stimuli used (simple oriented gratings vs. complex natural images), the current study design was very similar to that of Denison et al. (2016). Both studies involved statistical learning of four arbitrary triplet sequences followed immediately by a binocular rivalry test to assess how learning of these sequences created expectations that influenced visual perceptual selection. Neurons in early visual cortical areas exhibit orientation selectivity for simple edges (Hubel & Wiesel, 1962), while neural representations of complex object features like those in natural images have been found in higher-order ventral visual cortical areas (reviewed in Tanaka, 1996). Thus, the location in the visual cortical hierarchy at which multiple possible perceptual interpretations are resolved may determine whether predictive context enhances or suppresses perceptual selection of the expected image.

A recent study proposed a solution to the “Perceptual Prediction Paradox,” in which enhancement of brain responses to expected stimuli is apparently incompatible with prioritization of more informative and surprising stimuli (Press et al., 2020). The authors describe a model in which responses that are consistent with expectations are initially enhanced, followed by prioritization of unexpected surprising events. This model may provide a means of reconciling the findings of Denison et al. (2016) (greater perceptual selection of unexpected natural images) with those of the present study (greater perceptual selection of expected oriented gratings), given that gratings are likely to be represented and to rival with each other at earlier stages of the visual processing pathway, compared to complex natural images.

Moreover, while the oriented gratings that were employed in our study can be represented along a single featural dimension, natural image representation requires a feature space with many more dimensions, so the degree of difference between pairs of individual complex stimuli will be greater. Although an orientation orthogonal to what is expected might be maximally surprising within a one-dimensional orientation space (as in the current study), seeing an animal when an indoor scene was expected (as in Denison et al., 2016) potentially creates greater surprise that involves multiple feature dimensions. Further work is needed to investigate how the visual system implements predictions and responds to surprise for stimuli that can be described with a small number of feature dimensions versus those that cannot.

The perceptual enhancement of unexpected natural images reported in Denison et al. (2016) was only evident in the first half of the binocular rivalry test trials. This was explained as a dilution of statistical learning over time, as an unpredicted stimulus was presented to one eye during every rivalry trial. An alternative explanation is that this dissipation of the effects of prediction reflects habituation to the surprising, unexpected natural images during the rivalry test period in Denison et al. (2016), resulting in a decreased neural response to surprise. In the current study, the degree of surprise due to unexpected orientations may not have been large enough to elicit strong neural surprise responses. In this case, the facilitatory effect of expectation would remain consistent throughout the binocular rivalry test trials, as we observed.

Early visual cortex and higher-order ventral visual cortex have also been reported to differ in the effects of prediction on representations of expected versus unexpected stimuli. In cortical area V1 in humans, expected orientations elicited a smaller functional MRI (fMRI) response than unexpected orientations, but performance of a classifier trained to discriminate patterns of activity associated with the orientations was greater for expected than unexpected stimuli (Kok et al., 2012). These effects of expectation on fMRI responses in early visual cortex were weaker in voxels that preferred the expected orientation compared to those that preferred the unexpected orientation, consistent with a sharpening effect of expectation on the selectivity of stimulus representations in cortical area V1. In addition, the amount of enhancement of classifier performance with expectation was correlated with better orientation discrimination for expected compared to unexpected stimuli, as assessed psychophysically (Kok et al., 2012).

However, both human fMRI (Richter et al., 2018) and macaque electrophysiological (Meyer & Olson, 2011) studies in higher-order ventral visual cortex showed that the magnitude of suppression of responses to expected versus unexpected images of objects scaled with the preference of neurons for the expected image, a relationship that is consistent with a dampening account and in the opposite direction of that reported by Kok et al. (2012) in early visual cortex (Richter et al. 2018) reported no conclusive evidence for either sharpening or dampening of fMRI representations in early visual cortex). In addition, although Kok et al. (2012) found enhanced classification performance for fMRI responses to expected stimuli in human early visual cortex, the opposite result was obtained in neural recordings in macaque inferior temporal cortex (Kumar et al., 2017).

These discrepant findings suggest that expectation may cause a relative enhancement of responses to expected stimuli in early visual cortex through sharpening and a relative suppression of responses to expected stimuli in higher-order visual cortex through dampening, a pattern that would be consistent with the different perceptual effects of expectation found in Denison et al. (2016) and the present study. Interestingly, repetition suppression effects have been reported to be independent in macaque V2 and inferotemporal cortex, based on differences in spatial specificity and temporal dynamics (Williams & Olson, 2022).

### Neural correlates of perception during binocular rivalry

Electrophysiological and fMRI signals correlate with alternations in perception during binocular rivalry in multiple visual areas, including the lateral geniculate nucleus (Haynes & Rees, 2005; Wunderlich et al., 2005), cortical area V1 (Leopold & Logothetis, 1996; Polonsky et al., 2000; Haynes & Rees, 2005), extrastriate visual cortical areas (Logothetis & Schall, 1989; Moutoussis et al., 2005), and ventral visual cortical regions (Sheinberg & Logothetis, 1997; Tong et al., 1998).

The proportion of neurons with responses that correlate with perception in binocular rivalry increases along the visual hierarchy. Neurophysiological studies in nonhuman primates have demonstrated that responses of most neurons in object-specific inferotemporal cortex correspond with the animal's perceptual report during rivalry (Sheinberg & Logothetis, 1997), but the proportions of such neurons are lower in earlier visual cortical areas like MT and V4 (Leopold & Logothetis, 1996; Logothetis & Schall, 1989), even lower in V1 and V2 (Leopold & Logothetis, 1996), and not detectable in the lateral geniculate nucleus (Lehky & Maunsell, 1996; Wilke et al., 2009). These differences along the visual pathways in the proportions of neurons that reflect conscious perception during binocular rivalry may also relate to the different directions of predictive effects on perceptual selection for natural images (Denison et al., 2016) versus oriented gratings (the current study).

### Effects of predictive context on latency and duration of initial percepts in binocular rivalry

Several studies have demonstrated a dissociation between effects of predictive context on the identity and on the duration of the initial percept in binocular rivalry (Denison et al., 2011; 2016; Piazza et al., 2018), and this is consistent with previous work indicating that different processes underlie perceptual selection and maintenance in rivalry (Bressler, Denison, & Silver, 2013; de Jong, Knapen, & van Ee, 2012; Levelt, 1965; Sobel & Blake, 2002; Stanley et al., 2011). In the present study, the predicted orientation was most likely to be initially perceived, was quicker to reach dominance, and had a shorter duration, compared to the unpredicted orientation.

Relatively shorter durations for initial perception of expected orientations may be due to capture of exogenous attention by the unexpected orientation. Unexpected stimuli are known to capture exogenous attention (reviewed in Carrasco, 2011), and exogenous feature-based attention increases initial dominance in binocular rivalry (Chong & Blake, 2006; Mitchell et al., 2004). Moreover, natural scenes with embedded incongruous objects are more likely to be initially dominant in binocular rivalry, compared to congruous objects (Mudrik, Breska, Lamy, & Deouell, 2011), and they also have extended periods of perceptual dominance (Mudrik, Deouell, & Lamy, 2011). However, unexpected natural images were found to be more likely to be initially dominant than expected images, with no significant difference in duration of dominance (Denison et al., 2016). In the current study, expected orientations were more likely to be initially selected, with a shorter latency, than unexpected orientations, but on trials in which the unexpected orientation was initially perceived, attention may have been drawn to this unexpected stimulus, thereby prolonging its duration of dominance.

### Future directions

We have shown that observers are more likely to perceive the expected grating orientation during binocular rivalry. This is in agreement with results from other studies of prediction and perceptual selection in binocular rivalry (Denison et al., 2011; Piazza et al., 2018), but it conflicts with the findings of a similar study using natural images (Denison et al., 2016). A promising direction for future research is to characterize how prediction influences perception and brain responses in intermediate areas of the ventral visual stream, using sequences of stimuli that drive V4 (e.g., contours [El-Shamayleh & Pasupathy, 2016] or non-Cartesian gratings (David et al., 2006) or mid-level textures (Long et al., 2018), including the study of individual differences in brain–behavior correlations for effects of prediction. It will also be important to differentiate predictive effects of enhancement versus suppression for visual perception and for brain responses to visual stimuli (Feuerriegel et al., 2021).

## Conclusion

We found that during binocular rivalry, orientations that were expected based on statistical learning of sequences were preferentially selected for conscious awareness. This finding is consistent with several previous studies but inconsistent with previous work that used a similar paradigm but with natural images. Whether perceptual selection of a given expected stimulus is enhanced or suppressed may depend on the level of the visual processing hierarchy at which perceptual inference occurs.
